# Heavier loads in flywheel exercise induce greater post-activation performance enhancement in countermovement jumps compared to heavy Smith machine squats in males

**DOI:** 10.5114/biolsport.2024.139075

**Published:** 2024-04-25

**Authors:** Jianhua Shi, Bing Yan, Mengjie Yu, Zhe Wang, Yang Wang, Haoyang Liu, Wei Zhang, Olivier Girard

**Affiliations:** 1 China Institute of Sport and Health Science, Beijing Sport University, Beijing, China; 2 School of Physical Education, Yanching Institute of Technology, Langfang, China; 3 Key Laboratory for Performance Training & Recovery of General Administration of Sport, Beijing, China; 4 Sanya Branch School of High School Affiliated Renmin University, Sanya, China; 5 School of Sports Engineering, Beijing Sport University, Beijing, China; 6 School of Human Sciences (Exercise and Sport Science), University of Western Australia, Perth, Western Australia

**Keywords:** Flywheel, Post-activation performance, enhancement, Resistance exercise, Conditioning activities, Vertical jump ability, Back squats

## Abstract

We evaluated the effects of post-activation performance enhancement through flywheel exercise with varying inertial loads compared to traditional resistance exercise on countermovement jump performance and muscle recruitment. In a randomized crossover design, 13 trained men completed four main experimental trials after three familiarization sessions. These conditions included a traditional trial consisting of 5 sets of 1 repetition using the Smith machine (SM) squat at 90% 1RM, and three flywheel ergometer trials. Each flywheel protocol consisted of 3 sets of 8 repetitions with 3-minute rest intervals between sets, utilizing one of three inertial loads (0.0465, 0.0784, and 0.1568 kg · m^2^ for light, moderate, and heavy, respectively). Participants performed countermovement jumps before (baseline), immediately after (0 minute), and at the fourth (+4 minutes), eighth (+8 minutes), and twelfth (+12 minutes) minute following exercise. Compared to baseline, jump height was higher at +4 minutes for SM squats (p = 0.009). All flywheel conditions exhibited higher jump heights at +4 minutes (p < 0.05), +8 minutes (p < 0.001), and +12 minutes (p < 0.001) compared to baseline. Additionally, moderate and heavy loads resulted in higher jump heights at 0 minute (both p < 0.001). Integrated electromyographic activity values, a proxy for muscle recruitment, were significantly higher for the *gluteus maximus* muscle at both +8 minutes and +12 minutes for moderate (both p = 0.004) and heavy loads (p ≤ 0.002) compared to SM squats. Overall, flywheel protocols produce greater post-activation performance enhancement, extend the time window for improvement, and recruit more active musculature compared to heavy-load SM squats, particularly with heavier loads acting as a stronger preload stimulus.

## INTRODUCTION

Post-activation performance enhancement (PAPE) refers to a significant increase in an athlete’s power output shortly after a preconditioning activity, typically occurring within 4–12 minutes; however, earlier or later potentiation responses have also been observed [1]. Importantly, PAPE should not be confused with post-activation potentiation, which pertains to increases in twitch forces evoked by prior muscle activity and phosphorylation of myosin regulatory light chains [1, 2]. PAPE is commonly incorporated into pre-competition or pre-training warm-up routines to optimize athlete performance. This phenomenon is often attributed to the enhancement of the neuromuscular state of athletes, resulting in increased power output and subsequently improved sprint and/or vertical jump performance [2]. Nevertheless, it is widely acknowledged that an athlete’s performance in a countermovement jump (CMJ) depends on the contraction speed of the working muscle groups and the maximum force generated during the eccentric phase [3]. There is substantial evidence to suggest that single sets of heavy back squats (i.e., 80–90% 1RM [4]), also referred to as traditional protocols, can effectively induce PAPE during explosive movements [5]. However, alternative conditioning activities in the form of eccentric-overload resistance exercise have emerged as an alternative method to traditional protocols [6, 7].

Flywheel training has gained popularity as an approach to intensifying the eccentric phase of exercises compared to traditional weightstack exercise, promoting significant skeletal muscle adaptations [8]. Isoinertial devices, also known as flywheel ergometers, provide an accentuated eccentric muscle action and have proven effective in enhancing stretch-shortening cycle performance, thereby positively impacting explosive whole-body movements including jumping and sprinting [9]. Previous research has demonstrated that several weeks of inertial resistance training significantly increase the maximum strength of major muscle groups during the eccentric phase, leading to improvements in athletes’ vertical jump performance [8]. inertial flywheel resistance training stands out for its short familiarization period, even for inexperienced athletes [10]. Additionally, the portability of flywheel devices (typically weighting < 15 kg) has contributed to their growing popularity.

In recent years, there has been a substantial increase in studies exploring the effects of PAPE on vertical jumps (CMJ [11] and squat jumps [12]) using flywheel eccentric overload exercises (e.g., squat). As reviewed by Beato et al. [11], the volume (number of sets) and intensity (inertia) can be manipulated to influence the balance between neuromuscular potentiation and fatigue, which in turn determine the onset and magnitude of the PAPE response. For instance, a minimum of two sets of flywheel eccentric overload half-squats is required to enhance peak power responses during CMJ, even if no differences were observed in jump height, in male university soccer players [13]. Additionally, practitioners can use different inertia intensities (e.g., 0.03–0.11 kg · m^2^; [14]). However, most studies have primarily examined a single load when assessing PAPE time windows and magnitudes, and some have omitted reporting the intensity [7, 12], resulting in uncertainty regarding the effects of load intensity.

The effects of different flywheel inertial load intensities on PAPE in vertical jump performance have recently come under scrutiny [14, 15]. In a recent study by Fu et al. [15], the magnitude of PAPE in CMJ was influenced by the degree of inertial loads applied with increasing inertial loads (0.041, 0.057, and 0.122 kg · m^2^, respectively). On the contrary, there were no PAPE differences between small and large loads (0.029 *vs*. 0.061 kg · m^2^ [16]) or between medium and large flywheel loads (0.029 *vs*. 0.061 kg · m^2^ [14]) on subsequent athletic performance. More research on the dose-dependent effects of flywheel conditioning intensity on PAPE is necessary and can provide valuable insights into prescribing the appropriate intensity to acutely enhance explosive performance [11].

Surface electromyography (EMG) can be employed to assess the underlying neural mechanisms contributing to improvements in mechanical variables during CMJ [17]. Despite growing research determining the PAPE effects of flywheel exercise [18], there is a scarcity of studies that have examined specific muscle activity levels (i.e., measured by surface EMG) during movements. The execution of flywheel exercise across a range of inertial loads has produced conflicting results regarding motor unit recruitment. Some studies report modifications in motor unit recruitment with graded inertial loads [19], while others indicate no noticeable effect [20]. One important shortcoming in the current PAPE literature is the absence of studies comparing surface EMG data after completing flywheel and gravity-based load protocols. To date, there is a lack of time-course and magnitude data available regarding the muscle recruitment strategies that occur during jump execution following flywheel exercise, notably when comparing different inertial loads.

The aim of this study was to compare the impact of flywheel resistance exercise with varying inertial loads to traditional resistance training on PAPE and the accompanying muscle recruitment during the CMJ. We hypothesized that flywheel exercise, especially with heavier loads, would enhance CMJ performance by increasing motor unit recruitment compared to traditional resistance exercise. The significance of this study is underscored by the observation that the majority of the fifty-one practitioners (~84%) surveyed by de Keijzer et al. [21] ‘agreed’ or ‘strongly agreed’ that *“isoinertial protocols can acutely enhance (PAPE) sport-specific performance”*.

## MATERIALS AND METHODS

### Participants

An *a priori* power analysis (α = 0.05, 1-β = 0.95) was performed using G*Power (Version 3.1.9.7) for the change in vertical jump height. The power calculation considered an ANOVA with repeated measures, involving four groups and five time points, considering a within-between interaction (correlation among repeated measures: 0.5). The averaged effect size for an acute change in vertical jump height, derived from expected performance improvement occurring from baseline to +4 minutes post-conditioning routine using moderate-to-large inertia levels (0.057–0.122 kg · m^2^), is 0.94 [15]. To express our results with 95% confidence, a minimum sample size of 8 participants was obtained, with ten allowing for a 20% attrition rate. Therefore, we recruited thirteen males, each with at least two years of resistance training history (mean ± SD, training history: 4.7 ± 0.9 years; height: 177.4 ± 2.5 cm; body mass: 73.6 ± 8.2 kg). Participants were classified as ‘Highly trained/National level’ (Tier 3) using established criteria [22] and were active in different sports (i.e., basketball, volleyball, track and field). They reported a two-year history of resistance training, including at least two sessions per week, and demonstrated experience in the back squat, with a minimum 1RM of 1.5 times their body mass (back squat 1RM: 141.5 ± 27.8 kg). The study was approved by the local ethics committee, and complied with the principles of the Declaration of Helsinki, with written informed consent obtained from participants.

### Study design

This study employed a randomized, cross-over design. Participants attended the laboratory on eight separate occasions over a 9-week period. The sessions included one preliminary testing session (visit 1), three familiarization sessions (visits 2–4) in line with the recommendation of Sabido et al. [10], and four main experimental trials (visits 5–8). All sessions were conducted at the same time of day (± 1 h) and separated by at least 48 hours [23].

The preliminary testing session (~60 minutes) aimed to collect biometric data, explain testing procedures, and complete 1RM back squat and CMJ assessments. During familiarization visits (~30 minutes each), participants familiarized themselves with study procedures including CMJ test, flywheel operation and PAPE protocol. During each visit, they practice flywheel resistance exercise procedures, which included three sets of eight repetitions with Light (FL), Moderate (FM), or Heavy (FH) inertial loads (visits 2, 3, and 4, respectively). During the four main sessions (visits 5–8), participants either performed a traditional trial consisting of five sets of one repetition using the barbell back squat (BS) with 90% 1RM or one of three inertial flywheel trials. Each flywheel trial consisted of three sets of eight repetitions, utilizing one of the three inertial loads. During experimental trials, three CMJ attempts were performed before (Baseline), immediately after (0 minute), and at the fourth (+4 minutes), eighth (+8 minutes), and twelfth (+12 minutes) minute following each conditioning routine. Participants were provided 30 seconds of rest between each CMJ attempt, and the best of three trials (jump height) was recorded for subsequent analysis.

Participants were instructed to avoid alcohol and caffeine consumption and to refrain from strenuous training in the 24 h prior to each session. They were also asked to maintain a 24-hour food diary before testing and replicate their diet before the four trials. During the testing period, they were instructed to maintain their normal diet, avoiding the use of nutritional supplements. Each trial had a duration of ~35 min, and the air temperature was maintained at a constant level around ~22°C.

### Experimental trials

**FIG. 1:**
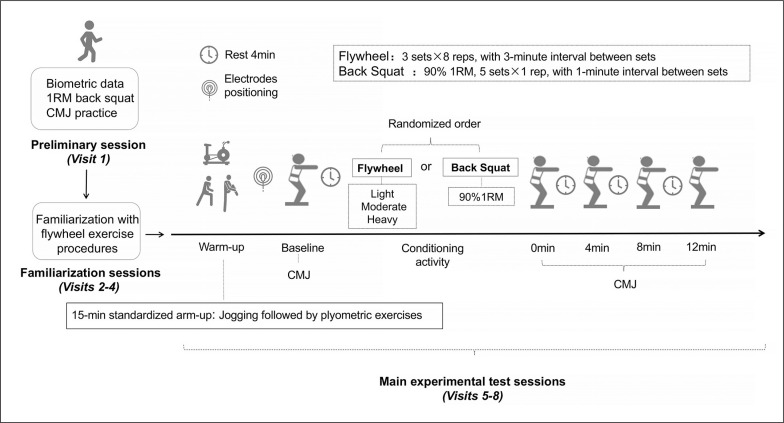
Experimental overview The four conditions include conditioning with a traditional trial, which consists of five sets of one repetition using the barbell squat BS) with 90% 1RM, or one of three inertial flywheel trials. Each flywheel trial consists of three sets of eight repetitions, utilizing one of the three inertial loads (Light, Moderate, Heavy).

The structure of the experimental visits is depicted in Figure 1. Upon arrival at the laboratory, participants completed a 15-minute standardized warm-up, which included jogging followed by plyometric exercises. After five minutes of passive seated rest, they underwent baseline testing for CMJ and then proceeded with a conditioning routine in one of the four experimental conditions.

In the traditional barbell protocol, subjects using the Smith machine perform a barbell BS with 90% 1RM, which consists of five sets of one repetition, with a 1-minute rest interval between sets.

In the inertial flywheel protocol, participants utilized a flywheel device (D11 Full, Desmotec, Biella, Italy) equipped with various inertial loads (0.0465, 0.0784, and 0.1568 kg · m^2^ for FL, FM, and FH, respectively) for three sets of eight repetitions of the half-squat exercise, with 3-minute rest intervals between sets. Each participants assumed a half-squat position, with their thighs parallel to the floor, crossing their hands on their shoulders and positioning their feet shoulder-width apart. The first two repetitions served to initiate the movement [15]. Subsequently, the next six repetitions were executed with maximal effort, aiming for maximal possible concentric speed during the entire concentric phase, which ranged from a 90° knee flexion to near full extension [13]. Since inertial forces initiate the reversed eccentric action at the end of the concentric phase, participants had to exercise control during the initial one-third of the movement and then apply maximal force to stop the motion at ~90° knee flexion [8].

### Procedures One-repetition maximum testing

Before the test performed on a Smith machine, participants completed a brief warm-up protocol consisting of submaximal squats at 50, 70, 80, and 90% of their self-estimated 1RM. This entailed 10, 6, 3 and 1 repetition(s) for each percentage, respectively, with a 2-minute rest between sets. Then, the load was gradually increased in 4–5 trials with at least 3 minutes of rest between each trial until the participant reached their 1RM [24]. The squat depth was individually standardized to parallel, meaning that they descended to reach a 90° knee angle. All participants reached their 1RM within a maximum of five trials.

### Countermovement jumps

To perform the CMJ test, participants were instructed to jump as high as possible. They started from a standing position and performed a downward countermovement to a self-selected depth, followed immediately by a rapid jump in one continuous movement. Participants were instructed to keep their hands on their hips to eliminate any influence of arm swing. The jumping was performed while participants stood on a force platform, which allowed for direct measurement of the vertical ground reaction forces. Data were sampled at a frequency of 1,000 Hz (model 9286BA, Kistler Corporation, Switzerland). Vertical jump height, peak power output, and take-off velocity were all calculated using the MARS software (version 4.0; Kistler Corporation, Switzerland). Participants completed three trials of the CMJ, with a 30-second rest between each trial. For the final analysis, we utilized the best result from three trials, determined by vertical jump height.

### Surface electromyography

A wireless receiver (Noraxon Inc, USA) was used to record raw surface EMG signals during the CMJ test from the *gluteus maximus, rectus femoris*, and *biceps femoris* muscles at a sampling rate of 1,000 Hz using MyoResearch Version 3.12 software (Noraxon Inc, USA). Before electrode placement, the skin was prepared by shaving hair off the desired area, lightly abrading, and cleaning with an alcohol swab. Bipolar surface electrodes with an interelectrode distance of 30 mm were then attached to the skin, aligned parallel to the underlying muscle fibers. Raw EMG signals were processed offline using MyoResearch software V.3.12 with a bandwidth of 10–500 Hz. EMG signal segments selected and integrated (i.e., iEMG value) for analysis correspond to the muscles of the dominant leg during the concentric stage. Each value represents the maximal EMG response for CMJ at various time points under each condition, expressed in absolute terms (uV · s).

### Statistical Analyses

Values are expressed as means ± SD along with the mean difference and its 95% confidence interval. Two-way repeated-measures ANOVA (Condition [BS, FL, FM and FH] × Time [baseline, 0 min, +4 min, +8 min, and +12 min]) were used to compare dependent variables. Normal distribution of the residuals was tested using the Kolmogorov– Smirnov test. Data variance was first assessed using Mauchly test of sphericity, and a Greenhouse–Geisser correction was applied when required. Post hoc pairwise comparisons were performed using Bonferroni-adjusted P values. For each ANOVA, partial eta-squared ($\eta_{\text{p}}^{2}$, with $\eta_{\text{p}}^{2}$ ≥ 0.06 representing a *moderate* effect and $\eta_{\text{p}}^{2}$ ≥ 0.14 a *large* effect) were calculated as measures of effect size. Cohen’s *d* effect sizes were calculated to determine meaningful differences, with *d* < 0.2, *d* = 0.2–0.5, *d* = 0.5–0.8 and *d* > 0.8 representing *trivial, small, moderate* and *large* effect sizes, respectively. Dependent variables were expressed as the percent change (%) from the baseline measurements. Statistical significance was set at p ≤ 0.05.

## RESULTS

### Mechanical performance

**FIG. 2:**
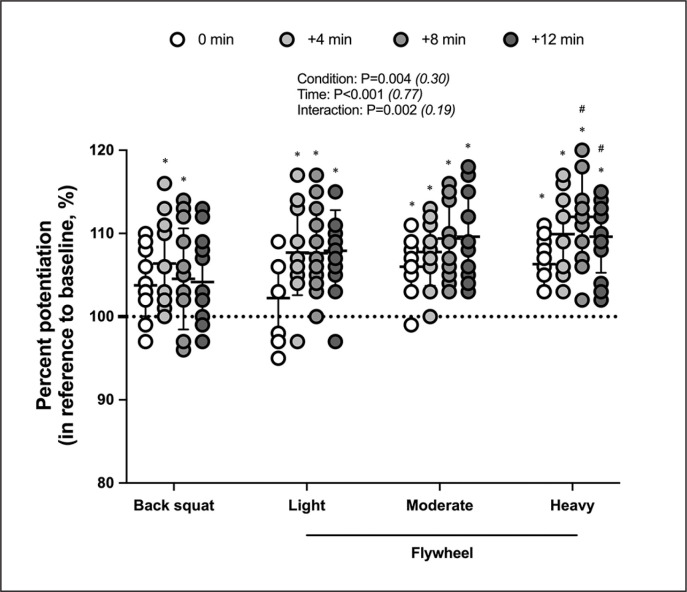
Individual data for percent potentiation (jump height) at various time points following four conditioning routines in reference to baseline. The four conditions include conditioning with a traditional trial, which consists of five sets of one repetition using the barbell squat with 90% 1RM, or one of three inertial flywheel trials. Each flywheel trial consists of three sets of eight repetitions, utilizing one of the three inertial loads (Light, Moderate, Heavy). Values are mean ± SD (n = 13). Note that values less than 100 indicate performance decrement, while values greater than 100 indicate performance benefits. * *P < 0.05, significantly different from baseline (assigned the value 100) in a given condition;* ^#^ *P < 0.05, significant different from back squat at the same time*.

Figure 2 displays percent potentiation (derived from jump height) individual data.

**FIG. 3:**
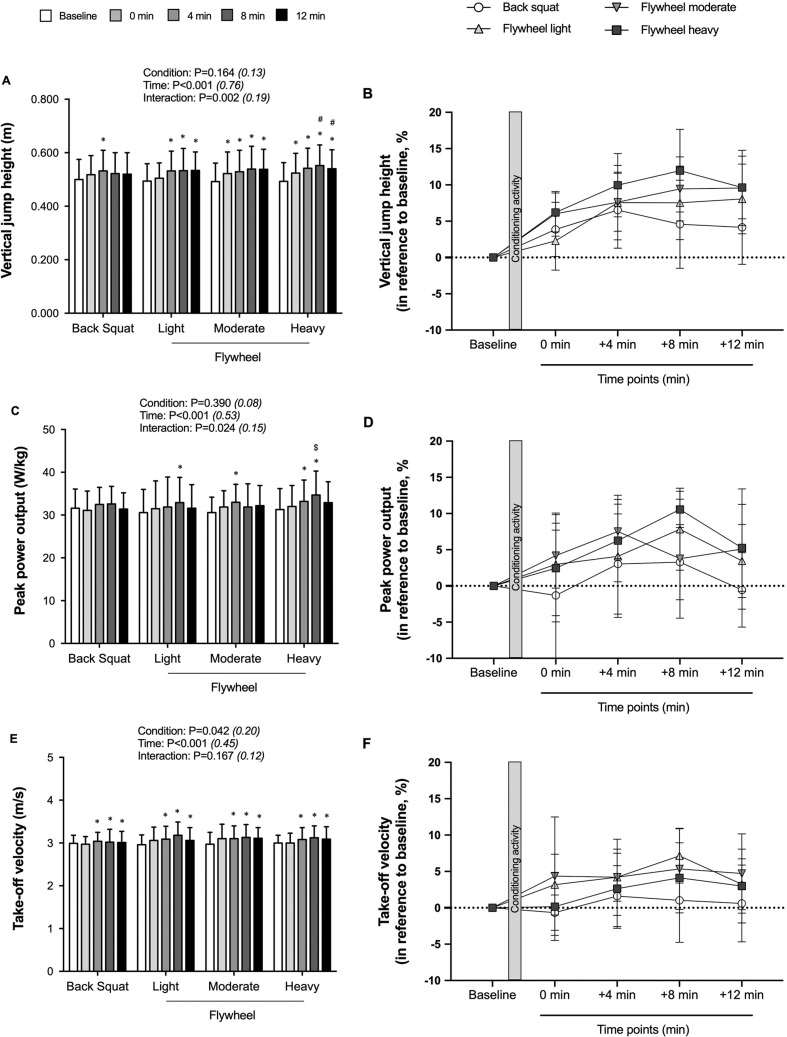
Mechanical variables (absolute values [left panels] and relative changes [right panels]) at various time points following four conditioning routines in reference to baseline. Changes in vertical jump height (A and B), relative peak power output (C and D), and vertical velocity at take-off (E and F). Values are mean ± SD (n = 13). The four conditions include conditioning with a traditional trial, which consists of five sets of one repetition using the barbell squat with 90% 1RM, or one of three inertial flywheel trials. Each flywheel trial consists of three sets of eight repetitions, utilizing one of the three inertial loads (Light, Moderate, Heavy). * *P < 0.05, significantly different from baseline in a given condition; ^#^ P < 0.05, significant different from back squat at the same time.* ^$^ *P < 0.05, significant different from flywheel moderate at the same time*.

Compared to baseline, vertical jump height was higher at +4 minutes for BS (p = 0.009; *d* = -0.42; -0.032 [-0.057; -0.007]) (Figure 3A-B). Regarding FL, vertical jump height values were higher at +4 minutes (p = 0.003; *d* = -0.54; -0.037 [-0.063; -0.012]), +8 minutes (p < 0.001; *d* = -0.51; -0.038 [-0.064; -0.012]) and +12 minutes (p < 0.001; *d* = -0.59; -0.040 [-0.061; -0.018]) *versus* baseline. For both FM and FH, vertical jump height values were higher at 0 minute (p < 0.001; *d* = -0.40; -0.030 [-0.047; -0.014] and p < 0.001; *d* = -0.43; -0.031 [-0.044; -0.017]), +4 minutes (p < 0.001; *d* = -0.51; -0.038 [-0.057; -0.019] and p < 0.001; *d* = -0.68; -0.049 [-0.069; -0.029]), +8 minutes (p < 0.001; *d* = -0.61 -0.047 [-0.070; -0.024] and p < 0.001; *d* = -0.80; -0.059 [-0.085; -0.033]) and +12 minutes (p < 0.001; *d* = -0.64; -0.046 [-0.070; -0.023] and p < 0.001; *d* = -0.67; -0.047 [-0.066; -0.027]) *versus* baseline. Higher vertical jump values were noted for FH at +8 minutes (p = 0.013; *d* = -0.38; -0.030 [-0.054; -0.006]) and +12 minutes (p = 0.031; *d* = -0.26; -0.020 [-0.038; -0.001]) in reference to BS.

Compared to baseline, peak power output was higher at +8 minutes for FL (p = 0.003; *d* = -0.42; -2.369 [-3.960; -0.778]), +4 minutes for FM (p < 0.001; *d* = -0.60; -2.331 [-3.471; -1.190]), and both +4 minutes (p = 0.017; *d* = -0.38; -1.892 [-3.504; -0.281]) and +8 minutes (p < 0.001; *d* = -0.63; -3.323 [-4.282; -2.364]) for FL (Figure 3C-D). Higher peak power output values were noted for FH at +8 minutes (p = 0.002; *d* = -0.38; -2.746 [-4.456; -1.037]) in reference to FM. Irrespective of condition, take-off velocity was higher at +4 minutes (p = 0.018; *d* = -0.41; -0.095 [-0.176; -0.014]), +8 minutes (p = 0.010; *d* = -0.53; -0.132 [-0.236; -0.028]), and +12 minutes (p = 0.044; *d* = -0.37; -0.086 [-0.169; -0.002]) in reference to baseline (Figure 3E-F).

**FIG. 4:**
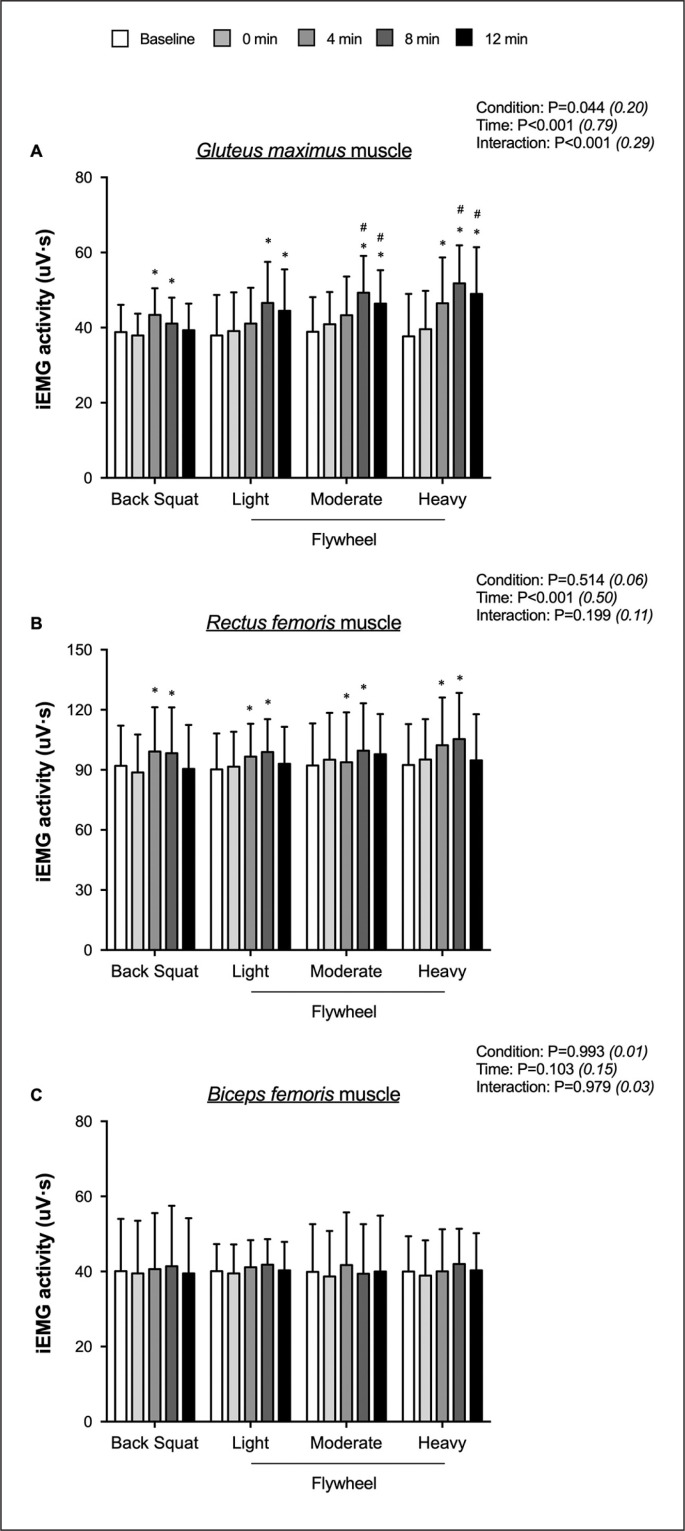
Integrated electromyographic (iEMG) activity for *gluteus maximus* (A), *rectus femoris* (B), and *biceps femoris* (C) muscles at various time points following four conditioning routines in reference to baseline. Values are mean ± SD (n = 13). The four conditions include conditioning with a traditional trial, which consists of five sets of one repetition using the barbell squat with 90% 1RM, or one of three inertial flywheel trials. Each flywheel trial consists of three sets of eight repetitions, utilizing one of the three inertial loads (Light, Moderate, Heavy). * *P < 0.05, significantly different from baseline in a given condition;* ^#^ *P < 0.05, significant different from back squat at the same time*.

### Surface EMG activity

For BS, *gluteus maximum* iEMG values were higher at +4 minutes (p < 0.001; *d* = -0.63; -4.562 [-6.462; -2.662]) and +8 minutes (p = 0.011; *d* = -0.33; -2.331 [-4.193; -0.468]) in reference to baseline. Compared to baseline, *gluteus maximum* iEMG values were higher at both +8 minutes and +12 minutes for FL (p = 0.001; *d* = -0.81 -8.731 [-13.937; -3.524] and p = 0.027; *d* = -0.61; -6.638 [-12.671; -0.606]) and FM (p < 0.001; *d* = -1.10; -10.392 [-15.128; -5.656] and p = 0.002; *d* = -0.83; -7.492 [-12.404; -2.580]). Regarding FH, *gluteus maximum* iEMG values were higher at +4 minutes (p = 0.011; *d* = -0.75; -8.800 [-15.858; -1.742]), +8 minutes (p < 0.001; *d* = -1.31; -14.100 [-20.659; -7.541]) and +12 minutes (p = 0.002; *d* = -0.95; -11.308 [-18.634; -3.981]) *versus* baseline. Higher *gluteus maximum* iEMG values were noted at both +8 minutes and +12 minutes for FM (p = 0.004; *d* = -0.96; -8.131 [-13.769; -2.493] and p = 0.004; *d* = -0.66; -7.115 [-12.042; -2.189]) and FH (p < 0.001; *d* = -1.23; -10.677 [-16.028; -5.326] and p = 0.002; *d* = -0.78; -9.769 [-16.062; -3.476]) in reference to BS. Irrespective of condition, *rectus femoris* iEMG values was higher at +4 minutes (p = 0.050; *d* = -0.33; -6.283 [-12.568; 0.003]), and +8 minutes (p = 0.006; *d* = -0.46; -8.846 [-15.468; -2.224]) in reference to baseline (Figure 4 A-C).

## DISCUSSION

### Flywheel protocols with varying loads

As hypothesized, the magnitude and time course of CMJ performance improvements varied depending on flywheel resistance, notably with earlier and more pronounced effects observed with higher inertial loads. Our findings partially align with literature on changes in CMJ performance following PAPE protocols that involve flywheel exercises [14]. In a recent study, Fu et al. [15] found that both moderate resistance (0.057 kg · m^2^) and heavy resistance (0.122 kg · m^2^) resulted in enhanced CMJ performance between 4 and 8 minutes, whereas no change was observed with light loads (0.041 kg · m^2^). However, in our study, PAPE was only evident with moderate and heavy inertial loads immediately after the conditioning activity. Contrastingly, when comparing moderate (0.03 kg · m^2^) and high (0.06 kg · m^2^) inertial flywheel half-squat intensities, Beato et al. [14] found no significant difference between the protocols regarding the onset and magnitude of the resulting PAPE effects (~10%) in relation to CMJ performance. Despite previous investigations and the current study using the same flywheel device for preconditioning purposes, discrepant findings could be primarily attributed to other methodological differences: 1) the exercise routine included either three sets of six repetitions with two minutes of rest [14] or four sets of seven repetitions with three minutes of rest [15] *versus* three sets of six repetitions with three minutes of rest (current study); 2) inertial loads ranging from 0.03–0.06 kg · m^2^ [14] or 0.041–0.122 kg · m^2^ [15] *versus* 0.046–0.157 kg · m^2^ (current study); 3) differences in training levels of tested individuals (participants in our study exhibited smaller values of concentric peak power compared to those in other studies [14]). Altogether, our findings suggest that when implementing flywheel exercise for PAPE, different inertial loads should not be used interchangeably.

### Wider time windows with flywheel protocols

Another novel finding was that flywheel protocols elicit PAPE in a shorter recovery interval compared to BS, with PAPE onset observed at 0 minute for FM and FH and +4 minutes for FL. Previous flywheel studies have shown either decreased [14] or increased [15] CMJ performance immediately after the conditioning activity. Our findings do not support the suggestion of a longer necessary time window between the conditioning activity (i.e., preload) and the subsequent performance test for fatigue to dissipate more quickly, and to achieve PAPE using flywheel compared to traditional protocols [25]. However, the optimal recovery time should be determined individually, as some individuals with light and moderate flywheel loads required more time to dissipate the effects of fatigue (Figure 2).

In our study, the greatest effects with a BS protocol were observed between 4 and 8 minutes, with no differences from baseline outside this interval. Indeed, the effects of BS were short-lived, disappearing after 8 minutes. This finding aligns with conclusions from Dobbs et al. [26], suggesting that the greatest effects on jumping ability are achieved with traditional PAPE protocols after a rest period of 3 to 7 minutes. In contrast, flywheel protocols resulted in a sustained elevation of jump height for 8–12 minutes, regardless of the inertia used, although the effects gradually diminished. Nonetheless, only heavy-load flywheel exercise demonstrated superior effects compared to BS at both +8 and +12 minutes. Previous research has noted that PAPE induced by a single bout of flywheel resistance exercise (0.083 ± 0.03 kg · m^2^) on CMJ can persist up to 20 minutes [27]. Remarkably, the positive effects of the heaviest load (FH) were consistent, as all thirteen participants exhibited percent potentiation values exceeding 100% at all time points. Overall, our analysis of the CMJ performance results reveals a broader time window for PAPE in the three flywheel conditions compared to BS, with even earlier effects with moderate and heavy loads.

### Surface EMG activity

One remarkable observation was the significantly earlier and larger muscle recruitment of the *gluteus maximus* muscle with increasing inertia levels after the preload. Previous studies have not documented muscle recruitment patterns during CMJs in response to flywheel protocols with varying inertial loads, making comprehensive comparisons with the existing literature difficult. However, since surface EMG activity changes in the *gluteus maximus* muscle tended to follow a similar trend as jump height, it is plausible that increased muscle recruitment may contribute to PAPE [19]. This could result in increased force production through larger muscle recruitment during the concentric phase. The transition from eccentric to concentric phases in flywheel exercises may trigger a more pronounced stretch reflex [28]. Pending confirmatory research, the energy stored in the eccentric phase during this transition may enhance the subsequent concentric action performance to a greater extent compared to traditional resistance exercises or flywheel protocols using lighter loads [11]. In our study, however, significant increases in take-off velocity at 4–12 minutes time intervals were comparable between all conditions.

Surface EMG findings indicate a selective increase in muscle recruitment with varying conditioning protocols. Although *rectus femoris* muscle activity was enhanced at both +4 minutes and +8 minutes, these changes occurred regardless of the condition. Additionally, changes in recruitment for the *biceps femoris* muscle remained non-significant in our protocol. This suggests that our hypothesis, which proposed that different inertial loads likely differentially activate lower limb musculature, is only partially confirmed. However, it is important to exercise caution when interpreting surface EMG findings since this technique can not differentiate between the number of motor units and their firing frequencies, and there is also an inherent risk of crosstalk from adjacent muscles [29]. Previous studies have reported differences in recruitment sequences during actual flywheel exercises performed with varying inertia levels, indicating that inertia plays a significant role in modulating muscle recruitment. For instance, while the lowest load (0.025 kg · m^2^) follows the proximal-to-distal principle of muscle activation, higher loads (0.05–0.01 kg · m^2^) result in a reorganization of the underlying muscle coordination mechanisms [19]. Future studies should expand the number of studied muscle and determine whether jumping movements may be reorganized in response to PAPE.

### Individual responses

Compared to BS, the range of individual percent potentiation values was relatively consistent across all time points for the other three conditions, regardless of the inertial load (Figure 2). For instance, percent potentiation values for at least two conditions (BS and FL) ranged from -5% (0 minute) to +15% (+12 minutes) at 0–12 minutes time intervals. Substantial inter-individual variability was noticeable, despite all participants having engaged in resistance exercise at least twice weekly over the previous two years. Given the variations in participants’ characteristics, there is no consensus on the optimal PAPE window between a conditioning activity and subsequent explosive activities. A study by Gourgoulis et al. [30] found that stronger athletes (squat weights > 160 kg) experienced a more substantial increase in CMJ height (4% *vs*. 0.4%) compared to weaker athletes (squat weights < 160 kg) after five sets of back squats. Further investigation is needed to determine if stronger participants consistently exhibit a significantly greater PAPE response than their weaker counterparts, possibly due to enhanced activation of fast motor units, at all post-conditioning time points. Therefore, it is reasonable to assume that the PAPE phenomenon may be subjectdependent and lacks optimal standards for both timing and intensity of the stimulus [11].

The wide inter-individual variation underscores the necessity to differentiate between average group responses and the individual diversity in responses to a given conditioning activity [31]. Rather than solely comparing group means at all time points, as done here and in most available studies [11], it is pertinent to also evaluate distinct individual responses to each conditioning protocol. In support of this approach, Scott et al. [32] demonstrated that individualized recovery intervals yielded unique responses for several outcome measures concerning baseline and between conditioning activities (hex bar deadlift and back squat). However, no PAPE response was observed when comparing CMJ variables at different recovery intervals (30 s, 90 s, and 180 s) to baseline measurements [32].

### Limitations and additional considerations

Our study is not without limitations. Firstly, adjusting the flywheel’s inertial setting during resistance exercise may have differential effects on force, power, and eccentric overload for men and women [33], implying that our conclusions should be limited to the male cohort under study. Secondly, greater concentric outputs during assisted squats lead to increased eccentric outputs and result in a higher mechanical load [34]. This raises the possibility that flywheel-assisted squats may generate a larger and longer-lasting PAPE effect in relation to CMJ. It also suggests that the necessary adjustments in terms of inertia and volume (number of repetitions per set and total number sets [11]), tailored to specific demographics, could be explored for optimal benefits, eventually considering speed-derived peak variables to demonstrate an eccentric overload [35]. In our study, it can not be excluded that a smaller number of repetitions in the conditioning with a traditional trial could, at least in part, explain the relatively lower benefits compared to inertial flywheel trials. Thirdly, most studies, including this one, typically involve exercises performed in a ‘traditional set’ structure where repetitions are executed consecutively without rest. To manage fatigue when lifting heavier loads or with larger volume (number of repetition) in conditioning exercises, an approach that deserves further attention is to use a ‘cluster set’ structure to maintain training quality [36], which could potentially enhance PAPE. Finally, this study did not evaluate peripheral adaptations (i.e., twitch responses from percutaneous nerve stimulation) that enable increased muscle responses after both flywheel and barbell squats protocols [37]. These adaptations could potentially account for changes in the balance between transient fatigue and potentiation, thereby explaining the substantial variability in the percent potentiation responses both within and between individuals [1]. Future studies should contemplate the integration of motor nerve or transcranial magnetic stimulation techniques in the realm of flywheel exercise to evaluate post-activation potentiation. This phenomenon, well-described with a short half-life (~28 s), amplifies muscle force production at submaximal levels of calcium saturation, corresponding to submaximal levels of muscle activation [1]. These electrophysiological techniques could provide valuable insights into the specific neuromuscular factors (i.e., post-activation potentiation) contributing to performance enhancement (i.e., PAPE) when modifying inertial load, even though twitch contractile enhancements have been noted without any observable PAPE (e.g., vertical jump height) [38].

### Practical implications

The data presented here support and emphasize that flywheel devices with a wide range of inertia levels can be used to induce PAPE. For practitioners seeking to acutely enhance their strength and power during training sessions or before competitions using this preload strategy, our findings suggest that higher inertias should be preferred. The limited time window for achieving an optimal PAPE response can be a challenge for strength and conditioning practitioners in real-world training scenarios, where time and access to heavy weights near the competition site are often limited. Our observations of a broader time window for PAPE in the three flywheel conditions compared to BS, with effects occurring even earlier with moderate and heavy loads, are highly relevant. In the context of boosting PAPE for CMJ using flywheel exercise, it appears that higher loads offer a better option.

## CONCLUSIONS

This study investigated the immediate effects of time intervals on CMJ performance and the corresponding muscle recruitment by comparing heavy-weight back squats with protocols involving flywheel exercise featuring varying levels of inertia. Flywheel exercise, irrespective of inertial loads, was more effective than back squats, as PAPE became apparent after 4–12 minutes, whereas with the traditional protocol, it was observed only after 4 minutes. Furthermore, there were immediate improvements in jump height following flywheel exercise, especially with moderate and heavy loads. Finally, EMG activity in the *gluteus maximus* and *rectus femoris* muscles (but not in the *biceps femoris*) increased in conjunction with PAPE, with the *gluteus maximus* mirroring the effects of inertial loads on jump height. Practically, the utilization of accentuated eccentric loading through flywheel exercise can serve as a strategy to enhance the recruitment of active musculature, potentially resulting in a more pronounced PAPE response [39]. Overall, the time window effect of PAPE after flywheel exercise differs from the traditional protocol in terms of CMJ performance and accompanying muscle recruitment. It demonstrates that flywheel protocols using heavier loads positively impact the magnitude of the PAPE response and the optimal time window.
